# Effect of Temperature and Stress on Creep Behavior of (TiB + TiC + Y_2_O_3_)/α-Ti Composite

**DOI:** 10.3390/ma18010110

**Published:** 2024-12-30

**Authors:** Xicheng Wang, Yunfei Zheng, Shiwei Han, Shulong Xiao, Jing Tian, Lijuan Xu

**Affiliations:** 1 National Key Laboratory for Precision Hot Processing of Metals, Harbin Institute of Technology, Harbin 150001, China; 24s009058@stu.hit.edu.cn (X.W.); hitzyf8193@163.com (Y.Z.); h1206299832@163.com (S.H.); xiaoshulong@hit.edu.cn (S.X.); tianjing@hit.edu.cn (J.T.); 2 School of Materials Science and Engineering, Harbin Institute of Technology, Harbin 150001, China

**Keywords:** hybrid reinforcement, creep behavior, reinforcements, silicide

## Abstract

In this study, a (TiB + TiC + Y_2_O_3_)/α-Ti composite was prepared by induction skull melting to investigate its creep behavior and microstructure evolution under different temperatures and stresses. The results show that the microstructure of the composite in the as-cast state is a basket-weave structure, and the main phase composition is α lamella, containing a small amount of β phase and equiaxed α phase. The creep life of the composite decreases significantly when the temperature is increased from 650 °C to 700 °C, and the steady-state creep rate is increased by 1 to 2 orders of magnitude. The creep stress exponent at 650 °C and 700 °C is 2.92 and 2.96, respectively, and the creep mechanism of the titanium matrix composite is dominated by dislocation movement. TiB and TiC exhibit synergistic strengthening effects, and Y_2_O_3_ remains stable during creep. The reinforcements strengthen the composite by impeding the dislocation movement. The accelerated dissolution of β phase is one of the major reasons for the decrease of creep properties of composite with increasing temperature and stress. Silicide precipitation was observed near the reinforcements and dissolved β-Ti, mainly in elliptical or short rod shapes, which pins dislocations and improves the creep performance of the composite. The results of this study can provide theoretical guidance and practical reference for the subsequent development and application of hybrid reinforced titanium matrix composites.

## 1. Introduction

Titanium matrix composites (TMCs) combine the merits of titanium alloys and ceramic compounds, exhibiting exceptional properties such as low density, high specific strength, high temperature resistance, and corrosion resistance. Consequently, they are widely used as structural materials, especially in aerospace, military, and other fields with significant application prospects [1,2,3]. The properties of TMCs are determined by the matrix, reinforcements, and the bonding strength between them. At present, the most widely used matrix is near-α titanium alloy, which demonstrates superior temperature mechanical properties, plasticity, and creep properties compared with other titanium alloys [4,5]. The near-α alloy is dominated by Ti-Al-Sn-Zr-Mo-Si alloy [6]. The reinforcements of TMCs mainly include ceramic phases and rare earth oxide phases with high melting points. TiB and TiC in ceramic phases have outstanding advantages and exhibit great bonding with matrix, so they are widely applied at present. Research by Kurita [7] shows that the discontinuous distribution of TiB whisker in Ti-6Al-4V can significantly improve the yield strength of the material. In addition, compared with large-sized whisker, the miniaturization of whisker can improve the plasticity of the material, while TiC can significantly improve the high-temperature bearing capacity of TMCs [8]. Among rare earth oxides, Y_2_O_3_ has the lowest density and a low thermal expansion coefficient, which can significantly improve the creep resistance of the material. Yue added nano-sized Y_2_O_3_ to T4822 and found that there were a large number of twins and dislocations around Y_2_O_3_, resulting in a 26% increase in the material’s strength [9]. Li [10] reported that the tensile yield strength of the titanium matrix composite reinforced with 2.0 wt% Y_2_O_3_ particles can reach 1300 MPa at room temperature, which is 54% higher than that of the titanium alloy matrix without Y_2_O_3_. A study has pointed out [11] that there is a synergistic effect between different reinforcements, and the effect of adding multiple reinforcements exhibits a more significant impact than that of adding of a single phase. There is a synergistic effect observed between TiC and TiB, and the best synergistic effect is achieved when the volume ratio between the two reinforcements is 1:1, resulting in the best strengthening effect on the material [12,13]. Han [14] demonstrated that the bonding between Y_2_O_3_ and the matrix after creep is better than that of TiB and TiC, so adding Y_2_O_3_ to the titanium matrix composites can further improve the stability of their high temperature performance.

Creep property is an important index to measure the properties of materials, which has important guiding significance for the application of materials. Material composition, temperature and stress all affect the creep behavior of materials. Silicon element has the effect of improving the creep performance of materials and will form a solid solution to silicide during the creep process [15]. Silicides have a pinning effect on dislocations, which greatly improves the creep properties of materials whose creep mechanism is dislocation movement [16]. At present, some researchers have studied the precipitation of silicides and their effects in the creep process of multi-component hybrid reinforced TMCs [17,18], but the effects of different temperatures and stresses on creep behavior and silicide precipitation remain unclear.

Based on the above discussion, further research is needed to investigate the effects of temperature and stress on the creep behavior of TMCs and the microstructure evolution during the creep process. In this article, a titanium matrix composite hybrid reinforced with (TiB + TiC + Y_2_O_3_) was prepared by induction skull melting. The high temperature tensile creep experiments were conducted at different temperatures and stresses to analyze the effects of temperature and stress on creep behavior and microstructure evolution during creep.

## 2. Materials and Methods

### 2.1. Materials Preparation

In this study, near-α alloy Ti-6Al-4Sn-8Zr-0.8Mo-1W-1Nb-0.25Si was selected as the matrix, 3 vol% (TiB + TiC) and 0.3 vol% Y_2_O_3_ were selected as reinforcements to prepare a (TiB + TiC + Y_2_O_3_)/α-Ti composite (Abbreviated as TMC) by induction skull melting with a water-cooled copper crucible, where the volume ratio of TiB and TiC is 1:1. The raw materials for the preparation of TMC are high-purity Ti, Al, Sn, Zr, and Si (Purity > 99%), and elements such as W, Mo, and Nb are added by the way of intermediate alloys, including Al-Mo alloy (50.5 wt% Mo), Al-W alloy (57.44 wt% W) and Al-Nb alloy (83.15 wt% Nb), which facilitate accurate batching and simultaneously reduce melting temperature. TiB and TiC are obtained by adding B_4_C powder (40–50 μm, Yingtai company, Shanghai, China) and C powder (10 µm, Gaoke company, Beijing, China) to the raw material, and by in situ reaction with Ti during melting (5Ti + B_4_C = 4TiB + TiC and Ti + C = TiC). Conversely, Y_2_O_3_ was obtained by directly adding nano-sized Y_2_O_3_ powder (100 nm, Keyou company, Shenzhen, China) to the raw material.

The melting process is completed at 1550–1600 °C for a total of 30 min and must be completed under the protection of argon to avoid liquid metal splashing or reacting with active gases, which would produce additional impurities. The powder raw materials are wrapped in aluminum foil, where C powder and other materials are put into the furnace for melting. Specifically, B_4_C and Y_2_O_3_ powder are first placed in the charging hopper and added to the furnace after the other raw materials are completely melted. After the melting is completed, it is necessary to continue stirring for 5–10 min before pouring to ensure the uniformity of the molten metal, and finally the molten metal completed by melting is poured into a metal mold preheated at 300 °C to obtain a cast ingot with a diameter of 50 mm and a height of 70 mm.

### 2.2. Experimental Procedure

**Figure 1:**
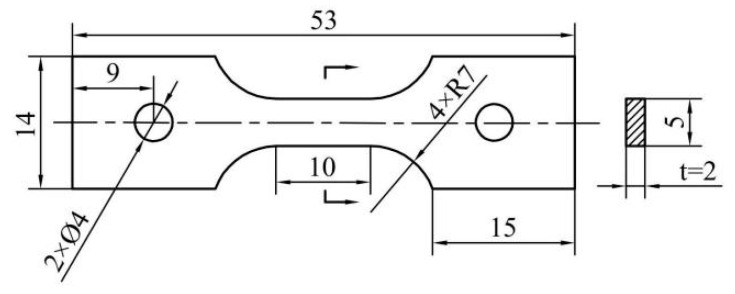
Dimensions of tensile creep specimens (in mm).

Firstly, used X-ray diffraction (XRD, X’PERT, Panalytical Company, Almelo, The Netherlands, Cu-K_α_) and scanning electron microscopy (SEM, Quanta 200FEG, FEI Company, Hillsboro, OR, USA, 20 kV) characterized the TMC in the as-cast state. And the microstructure and phases composition were observed and analyzed to ensure that the desired enhanced phases were obtained in the melting process. The specimens required for the experiment were cut from the ingot by electric discharge wire cutting. The size of the samples to observe microstructure were 10 mm × 10 mm × 5 mm, and the dimension of gauge segment of the specimens for the high temperature tensile creep experiment were 10 mm × 5 mm × 2 mm. The specific shape and dimension of the specimens are shown in Figure 1. The high temperature tensile creep tests were conducted on the RDL100 (Jiangsu Wallong-Hsin Machinery Engineering Corporation Ltd., Wuxi, China) tensile creep test machine at 650 °C and 700 °C, respectively, under experimental stresses ranging from 150 MPa to 200 MPa. Before the experiment, the surfaces of the specimens were polished with sandpaper to avoid the wire-cut defects affecting experimental results. Attach a thermocouple to each of the upper, middle, and lower parts of the specimen gauge segment to ensure the accuracy and stability of the temperature during the experiment. After the experiment, the fracture morphology of the fracture samples was observed and analyzed by SEM to study the creep fracture mode. For microstructural observation, cut off the deformed area of the sample and grind it to 3000 mesh sandpaper, then electrolytically polish and corrode with Kroll (3 vol% HF, 5 vol% HNO_3_, and 92 vol% H_2_O) solution; the deformed microstructure of the specimens was characterized by SEM. Select several representative specimens based on the scanning results, cut thin 300 μm slices using wire cutting, polish it to 50–60 μm by sandpaper, and then punch small circles with a diameter of 3 mm. Finally, use electrolytic double spraying to obtain thin areas for transmission electron microscopy (TEM, Talos F200X, Thermo Fisher Scientific, Waltham, MA, USA, 200 kV) characterization to further study the microstructure evolution of the composite. Compare the microstructure of the samples after creep under different temperatures and stresses to study the evolution of microstructure during creep and the impact of temperature and stress on the high temperature tensile creep behavior of TMCs.

## 3. Results and Discussion

### 3.1. Constituent Phase Identification and Original Microstructure

**Figure 2:**
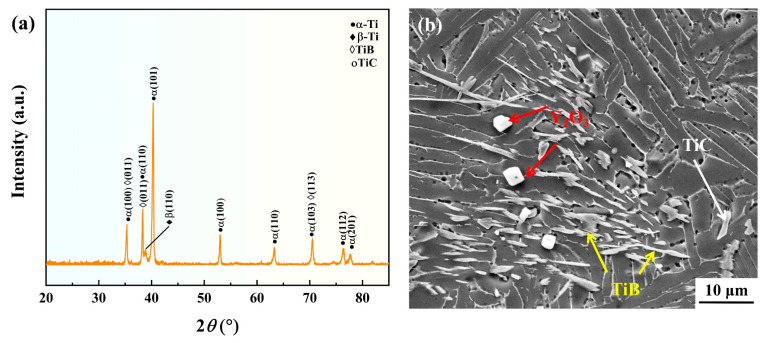
XRD pattern and SEM image of TMC: (**a**) XRD pattern; (**b**) SEM image.

To ensure that the added B_4_C powder and C powder can completely react with Ti to form TiB and TiC, without the generation of other side reaction products, the TMC in the as-cast state was characterized by XRD, the diffraction angle ranged from 20° to 90° with 0.02° step length, and the XRD pattern is shown in Figure 2a. The main phase composition of the TMC is α-Ti, with a relatively low of β-Ti. The diffraction peaks of TiB and TiC were found in the spectrum, while no diffraction peaks of raw materials or other products were observed, indicating that the reaction is complete and consistent with the expected results. Due to the addition of Y_2_O_3_ being too small, there is no diffraction peak in the spectrum.

The microstructure of TMC was further observed by SEM, and the SEM image was shown in Figure 2b. Obviously, it can be seen that the microstructure of the material in the as-cast state is a typical basket-weave structure. The reinforcements weaken the presence of the grain boundary α phase and promote the formation of basket-weave structure [17]. The main phases consist of TMC α phase (grayish white) and β phase (black); and α phase is predominantly lamellar, containing a small amount of equiaxed α phase. It can be seen that several reinforcements are evenly distributed in the matrix, and TiB grows fastest along the direction [010] [19], making it is easy to form a whisker shape with a relatively large aspect ratio. TiC is mainly equiaxed and distributed around TiB. Y_2_O_3_ is mainly micron-scale white granular or short rod-like, and the interface between Y_2_O_3_ and the matrix is very clean, without any reactant formation, which is the same as the situation in reference [20].

### 3.2. High Temperature Tensile Creep Properties

**Figure 3:**
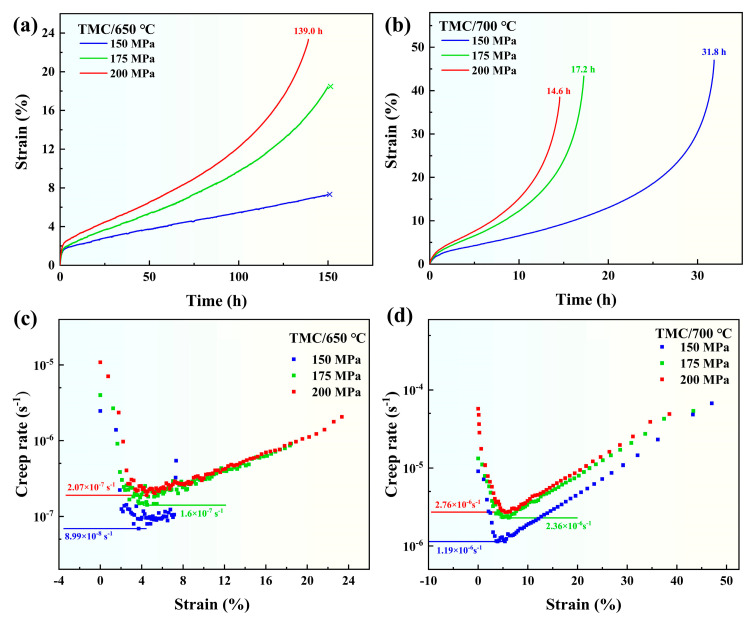
Creep curves and creep rate-strain curves of TMC at different temperatures and stresses: (**a**) creep curves under different stresses at 650 °C; (**b**) creep curves at different stresses at 700 °C; (**c**) creep rate-strain curves under different stresses at 650 °C; (**d**) creep rate-strain curves at different stresses at 700 °C.

Figure 3 shows the creep curves and creep rate-strain curves of TMC under different temperatures and stresses, in which the experiments are interrupted at 150 h under 650 °C/150 MPa and 650 °C/175 MPa, while the experiments were stopped when the specimen fractured under other conditions. Figure 3a,b shows the creep curves under different conditions. It can be observed from the creep curves that the creep of TMC has no obvious primary stage and quickly enters the steady state creep stage, and several specimens at 700 °C also enter the accelerating creep stage. The creep rate in the steady state creep stage can optimally represent the creep properties of material. According to the derivation of the creep curves, the creep rate was calculated and the creep rate-strain curves were made (Figure 3c,d). The experimental results indicate that when the temperature increases by 50 °C, the steady creep rate increases by 1 to 2 orders of magnitude, and the creep life decreases by 80%. With the increase of stress, the creep rate changes relatively little, indicating that the temperature exerts a considerable influence on the creep rate. Currently, creep research on titanium matrix composites is mostly conducted at temperatures below 650 °C [1,21,22]. Our material exhibits superior creep performance at 650 °C compared to other titanium matrix composites.

The steady-state creep rate can be expressed by the power law relationship [23]:

(1) $$\dot{\varepsilon }=A\sigma^{n}exp(-Q/RT)$$

In this equation, $\dot{\varepsilon }$ represents creep rate, *A* is a material dependent constant, *σ* represents creep stress (MPa), *n* is the creep stress exponent, *Q* is the creep activation energy (kJ/mol), *R* is a constant, and *T* is the Kelvin temperature (K).

**Figure 4:**
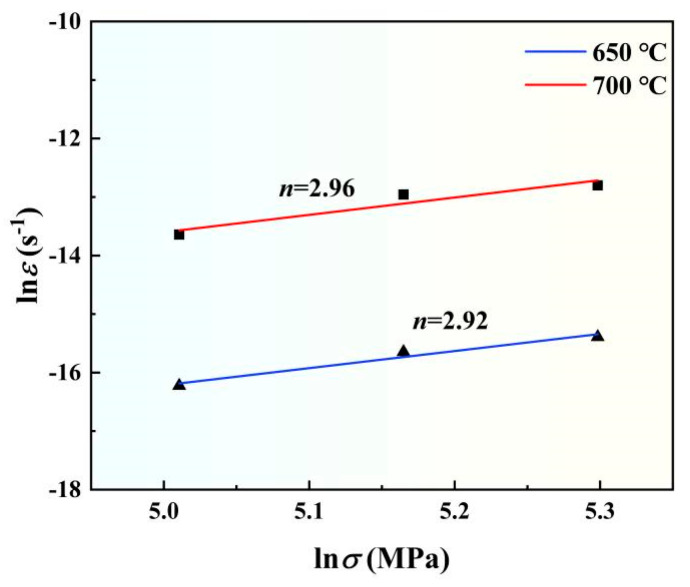
Logarithmic relationship between creep rate and stresses of TMC at 650 °C and 700 °C.

According to the data in Figure 3 and Equation (1), a double logarithmic plot illustrating the relationship between the creep rate and stress of TMC at 650 °C and 700 °C was made (Figure 4). The creep stress exponents of TMC at 650 °C and 700 °C were 2.92 and 2.96 respectively, both of which are close to 3, indicating that the creep mechanism of TMC is dislocations movement [24].

### 3.3. Creep Deformed Microstructure and Fracture Morphology

**Figure 5:**
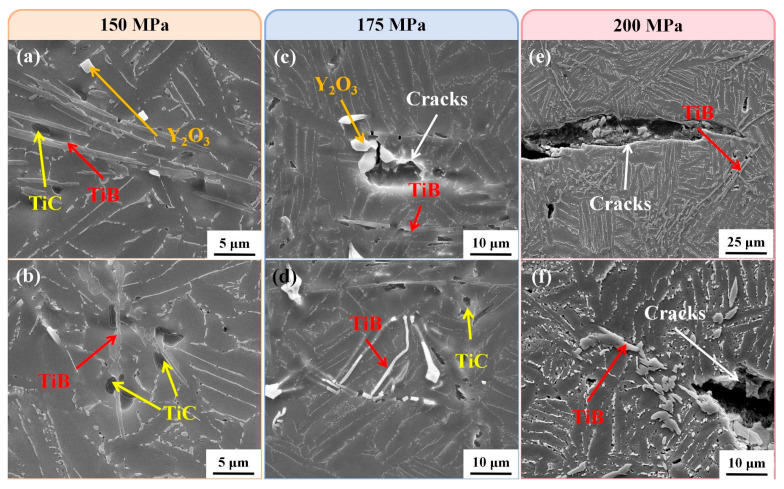
Creep deformation of TMC at 650 °C under different stresses: (**a**,**b**) 150 MPa; (**c**,**d**) 175 MPa; (**e**,**f**) 200 MPa.

Figure 5 shows the SEM characterization result of the deformation region of the specimens after creep under different conditions. As shown in Figure 5a,b, at 650 °C/150 MPa, TiB remain intact, while debonding occurs between TiC and the matrix. TiC precipitated separately is a branched structure, while TiB can be used as a heterogeneous nucleating particle of TiC. During solidification, TiC and TiB will form a symbiotic microstructure, as shown in Figure 5a,b. This microstructure effectively reduces the quantity of TiC branched crystals, resulting in TiC appearing as oval granular morphology and distributed near TiB [25,26]. The strengthening mechanism of discontinuous reinforcements is load transfer strengthening [27], and the TiB with a larger aspect ratio plays a more significant role in transfer strengthening [28]. Therefore, due to stress concentration TiB breaks first during the creep process. The interface between Y_2_O_3_ and the matrix is still intact after creep, and no debonding phenomenon occurs. This is because Y_2_O_3_ exhibits good thermodynamic stability and has a coefficient of thermal expansion similar to that of the matrix, leading to low interfacial stress between them during creep and maintenance of chemical stability. In composites, the location with the highest stress in the matrix is at the tip of the reinforcing phase, while the area with the highest stress within the reinforcing phase is its interior [29]. Y_2_O_3_ itself possesses high strength and is primarily in an equiaxed shape. The stress in the surrounding matrix is relatively low, insufficient to cause separation between Y_2_O_3_ and the matrix. Similarly, the internal stress is not enough to cause its destruction.

**Figure 6:**
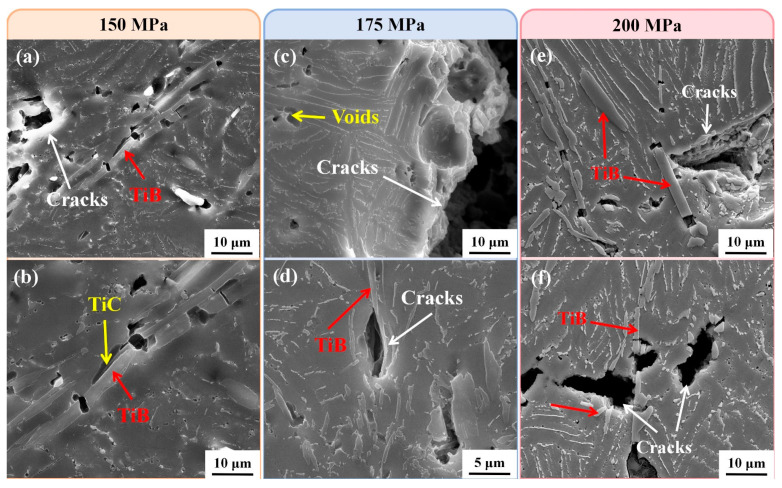
Creep deformation of TMC under different stresses at 700 °C: (**a**,**b**) 150 MPa; (**c**,**d**) 175 MPa; (**e**,**f**) 200 MPa.

Figure 6 shows the deformed microstructure after creep under different stresses at 700 °C. With the increase in temperature, the matrix softens, the load transfer makes the stress on the TiB more concentrated, which results in more severe fractures, and the cracks and voids in the deformed structure increase significantly. Moreover, the debonding between the matrix and TiB or TiC become more serious, indicating that the bonding strength between the two reinforcements and the matrix decreases further with the increase of temperature. In addition, high temperature may cause thermal expansion mismatch between the interface between the reinforcement and the matrix, resulting in an increase in interface stress, thus promoting the generation and expansion of cracks. According to Figure 5 and Figure 6, with the increase of stress, the deformation of the matrix intensifies, resulting in more pronounced stress concentration on the reinforcements. Consequently, the interface strength between the reinforcements and the matrix is lower than the individual strengths of these components. Thus, as stress continues to rise, the fracture of the reinforcements and the debonding between the reinforcements and the matrix become more severe. The number and dimension of cracks and voids along the reinforcements increase, and the cracks and voids gradually expand and connect during the creep process, ultimately causing specimen failures.

**Figure 7:**
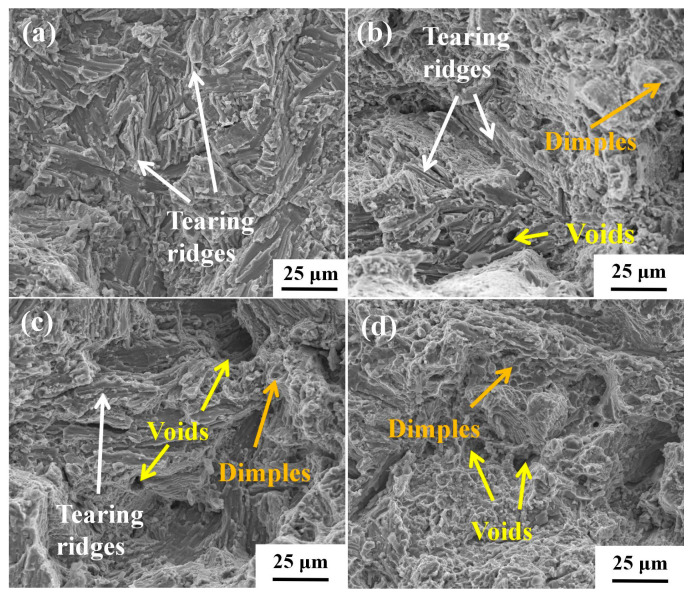
Fracture morphology after creep under different conditions: (**a**) 650 °C/200 MPa; (**b**) 700 °C/150 MPa; (**c**) 700 °C/175 MPa; (**d**) 700 °C/200 MPa.

Figure 7 shows the fracture morphologies of specimens after creep under different conditions. The specimens at 650 °C/150 MPa and 650 °C/175 MPa did not fracture. Comparing the conditions of Figure 7a 650 °C/200 MPa and Figure 7d 700 °C/200 MPa, it was observed that there were a large number of tearing edges existing in the fracture surface at the lower temperature, indicative that the fracture mode is brittle fracture. As the temperature increases, tearing edges decreases, while dimples increase significantly, and the fracture mode changes to mixed fracture. There are voids in Figure 7b–d, which are due to the stress concentration near the reinforcements during creep; and the bonding strength between the reinforcements and the matrix at high temperature is weaker than the strength of the reinforcements themselves. As a result, debonding occurs between reinforcements and the matrix under the action of stress and micropores. Compared with the fracture morphology under different stresses at 700 °C, it was observed that the fracture morphology has no obvious change with the increase of stress, indicating that the stress has little effect on the fracture mode.

### 3.4. Microstructure Evolution During Creep

**Figure 8:**
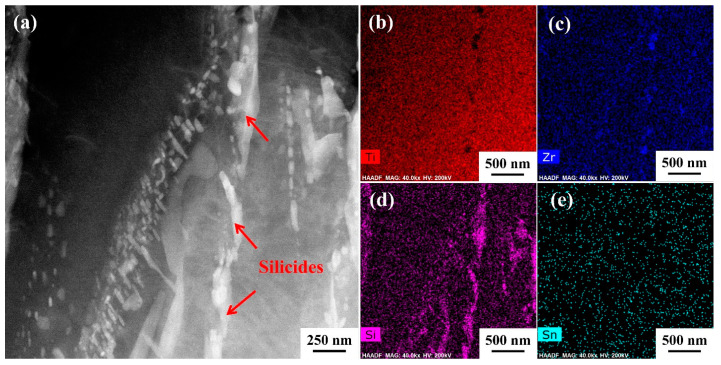
Precipitation and energy spectrum analysis of silicide after creep at 650 °C/150 MPa: (**a**) morphological distribution of silicides; (**b**–**e**) distribution of elements Ti, Sn, Zr, and Si, respectively.

As previously mentioned, the precipitation of silicide occurs during the creep process; other factors such as temperature [15] and element composition [30] will affect silicide precipitation. Figure 8 shows the TEM images of TMC specimens after creep at 650 °C/150 MPa. Figure 8a shows the morphology and distribution of the silicide precipitated, and Figure 8b–e shows the energy spectrum analysis of the corresponding region. It can be seen from the figures that the silicides precipitated under this condition are ternary compounds composed of Ti, Zr, and Si.

**Figure 9:**
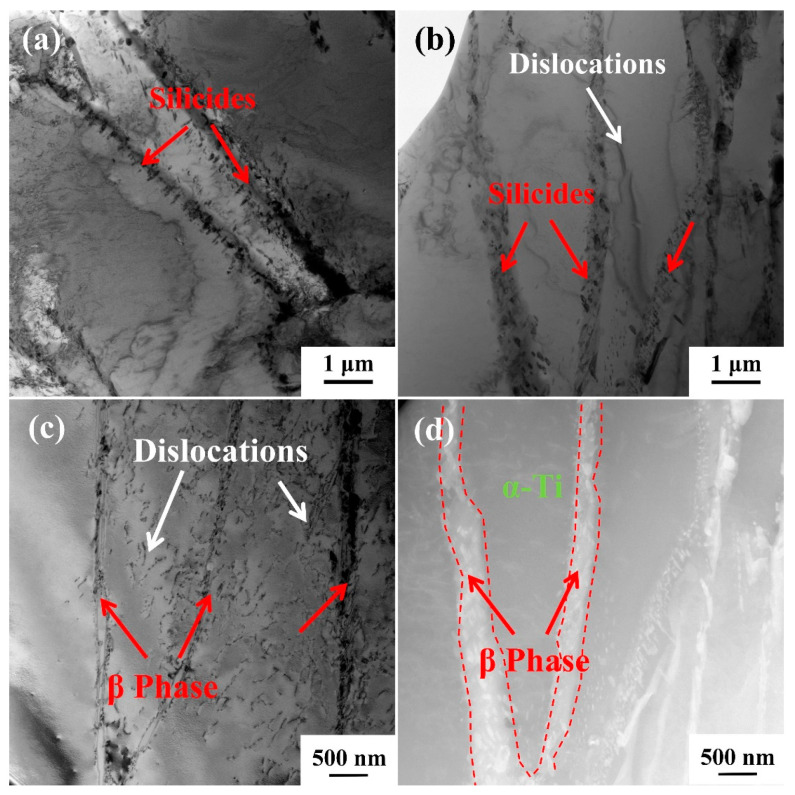
Microstructure evolution of TMC during creep at 650 °C/150 MPa: (**a**,**b**) morphological distribution of silicides and their pinning effect on dislocations; (**c**,**d**) dissolution of β-Ti and its adjacent dislocations.

Figure 9a shows that the silicides mainly exhibit a short rod-like morphology and are distributed around the α/β colonies. As shown in Figure 9b, silicide has a pinning effect on the dislocations, which effectively inhibits further expansion of the dislocation and thus improves the creep resistance of TMC [17,31]. Figure 9c,d show the dissolution of β-Ti during the creep process and the dislocations distribution around it. Due to the higher solubility of silicon in the β phases, the dissolution of β-Ti precipitates the enriched silicon elements, thereby promoting the precipitation of silicides, which is also the reason why silicides are mainly distributed around the α/β colonies. It is well known that the phase boundary has a hindering effect on dislocation motion, so the complete α/β colonies play a significant limiting role in the movement of the dislocations. During the creep process, the gradual dissolving of β-Ti leads to the α/β colonies being destroyed, and the limiting effect on dislocation movement is weakened, so the matrix will gradually soften and accelerate the fracture of the specimen.

**Figure 10:**
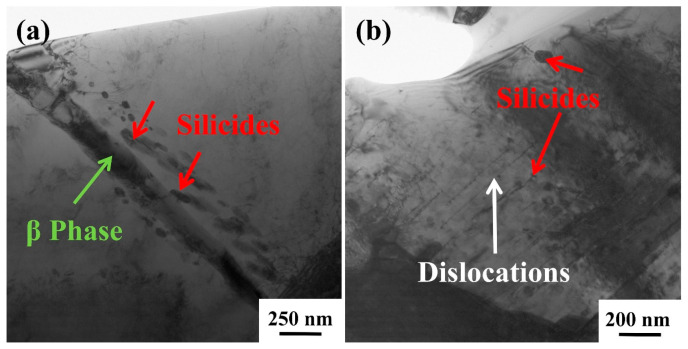
Dissolution of β-Ti and silicates precipitation of TMC after creep at 700 °C/150 MPa: (**a**) the dissolution of the β-Ti and its surrounding silicides; (**b**) the pinning effect of silicides on dislocations.

Figure 10 shows TEM images of the deformed microstructure of TMC after creep at 700 °C/150 MPa. Figure 10a shows the dissolution of β-Ti and the precipitation of silicides in its vicinity during creep. It is observed that the dissolution of β-Ti is exacerbated with the increase in temperature. This phenomenon can be attributed to the following: the dissolution of the β phase is a transition to the more stable α phase at high temperatures in titanium alloys. As the temperature rises, the diffusion rate of β-stabilizing elements like Mo in the β phase accelerates, reducing their concentration and making the phase transition more probable during creep. Consequently, temperature increase enhances the solubility of the β phase. The dissolution of the beta phase results in the restraining effect of α/β colonies on dislocation movement being weakened, leading to the creep performance of the material deteriorating accordingly. Consequently, the dissolution of β-Ti is one of the main reasons for the decrease in the creep properties of materials with increasing temperature. The dissolution of β-Ti resulted in a corresponding increase in the precipitation of silicides in the adjacent areas. Figure 10b shows that under the influence of silicides, the dislocations were basically parallel and the silicides had a pinning effect on the dislocations, causing them to stop expanding when they moved to the region near the silicides.

### 3.5. The Influence of Reinforcements on Microstructure and Creep Properties

**Figure 11:**
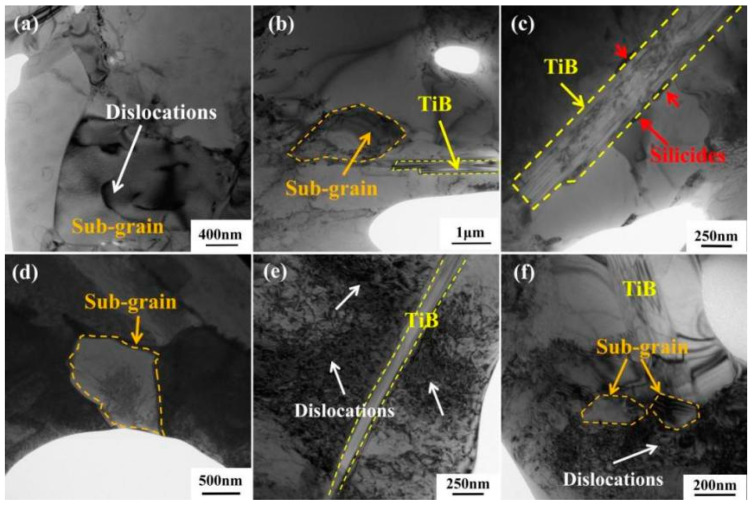
Morphology of the reinforcements after creep under different conditions: (**a**,**b**) 650 °C/150 MPa; (**c**,**d**) 700 °C/150 MPa; (**e**,**f**) 650 °C/200 MPa.

The influence of reinforcements on the creep properties of materials can be attributed to two aspects. On the one hand, the influence of the reinforcements on the microstructure of composites changes their creep properties. As mentioned earlier, the creep mechanism of the material in this study is dislocation movement, so the microstructure of the material has a very important influence on its creep properties. The addition of reinforcements such as TiB and TiC can change the microstructure of the material from the Widmanstatten microstructure to the basket-weave structure (see Figure 2b). Compared to the Widmanstatten microstructure, the basket-weave structure has better creep resistance, fatigue properties, creep properties, and plasticity [32,33]. Reinforcements such as TiB, TiC, and Y_2_O_3_ have the effect of refining grains [34,35,36], and it is worth noting that Y_2_O_3_ can also reduce the spacing of α laminates [37]. The reduction of grain size leads to a decrease in the effective slip length of dislocations, and creep resistance increases, thus improving the creep property of the material. In addition, these reinforcements facilitate the homogeneity of the material microstructure and improve its overall properties [38]. On the other hand, the reinforcements themselves play a role in improving the creep performance of the material. As shown in Figure 11e,f, during the creep process, the reinforcements can hinder the migration of dislocations and increase the dislocation density in their vicinity, thus improving the creep performance of the material. TiB plays a role in bearing loads and increases the creep resistance of materials.

Figure 11 shows the morphology of the reinforcements of TMC after creep at 650 °C/150 MPa and 700 °C/150 MPa, and the silicides and sub-grains near the reinforcements. Figure 11a,b show the morphology of TiB and its adjacent sub-grains after creep at 650 °C/150 MPa. The TiB can effectively hinder the migration of dislocations, causing the dislocations to accumulate near it. Due to the spontaneous reduction of energy, the high density of the dislocations will be rearranged and form sub-grains. Under this condition, dislocations still exist at the edge of the sub-grain, indicating that it is still in an unstable state [39]. Figure 11c shows that there are large amounts of silicides around TiB, and that TiB whisker accelerates the silicide precipitation behavior by hindering the migration of dislocations and providing nucleation sites for silicides [40]. Figure 11d shows the microstructure morphology of sub-grains after creep at 700 °C/150 MPa, where no dislocations exist at the edge, and it is already in a stable state, indicating that temperature increase accelerates the migration of dislocations and promotes dynamic recrystallization.

Reinforcement also has an adverse effect on the overall properties of composites. During the process of tensile fracture, cracks occur easily near the reinforcements, so the addition of the reinforcements leads to a decrease in the plasticity of the material [41]. In addition, as shown in Figure 11, the α phases around TiB are all equiaxial. Mohan [42] has shown that TiB can act as the heterogeneous nucleation sites of fine β-Ti in liquid titanium and eventually transform into equiaxed α grains. Feng [43] also found that α-Ti near TiB is prone to spheroidization behavior. The α/β colonies are semi-coherent [44], which helps to hinder the migration of the dislocations, while the equiaxial α and β interface is incoherent, and the dislocations move faster. Consequently, equiaxial α-Ti has a negative effect on the creep properties of the composites. In this study, the amount of TiB added was small, so the content of equiaxial α is small (as shown in Figure 3), and the influence on the creep properties of the composite is negligible.

### 3.6. Precipitation of Silicides

The silicon element in the composites will gradually precipitate to form silicide during creep process. The previous text pointed out that silicides are prone to precipitate near the dissolved β phase or the reinforcements. The density of dislocations near the reinforcements is elevated, and the dislocations can accelerate the diffusion rate of the elements, thus promoting the precipitation of silicides [45].

**Figure 12:**
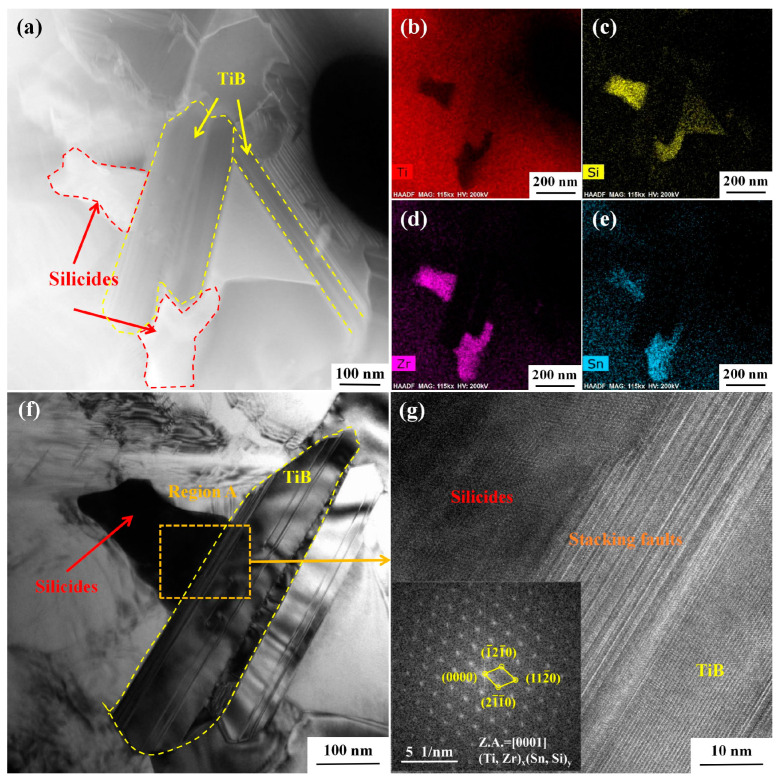
Silicides near TiB after creep at 650 °C/200 MPa: (**a**) TiB and nearby silicides; (**b**–**e**) spectrum analysis of the corresponding region in (**a**); (**f**) TiB and its surrounding silicides; (**g**) high-resolution images of TiB and silicides and diffraction spots of silicides.

Figure 12 shows the silicides near TiB after creep at 650 °C; both ellipsoidal or rod-like S2 silicides (Figure 8, Figure 9 and Figure 10) and large-size equiaxed silicides (Figure 12) were observed. The presence of Zr element can promote the precipitation of silicide, as Zr is a β-stable element. Moreover, the affinity between Si and Ti is lower than that between Si and Zr [31]. Therefore, some Ti elements are replaced by Zr during the formation of silicides, and the silicides precipitated at 650 °C/150 MPa are Ti, Zr, and Si ternary silicides. In addition, Zr has the effect of refining silicides [46].

With the increase in stress, the diffusion rate of atoms is significantly accelerated, which is conducive to the growth of silicides, so the size of silicides formed after creep at 650 °C/200 MPa is significantly larger than that after creep at 650 °C/150 MPa (Figure 12). According to the energy spectrum analysis in Figure 12, a portion of Si of the silicide precipitated at 650 °C/200 MPa is replaced by Sn to form quaternary silicide. The high-resolution image and diffraction spots of the silicide are shown in Figure 12g. The silicide has a close-packed hexagonal structure, and the direction (2$\bar{1}\bar{1}$0) of the silicide is parallel to the direction (010) of the TiB. With the increase in temperature, the dissolution degree of β phase increases at 700 °C, and the silicide precipitated near it increases correspondingly. As the temperature rises, the atomic diffusion rate accelerates, and the generation and movement of dislocations also speed up. This leads to the easier formation of high-density dislocations and accelerated element diffusion, which in turn promotes the precipitation of silicides [47]. Therefore, higher temperatures tend to facilitate the formation of larger-sized silicides. Kartamyshev [48] pointed out that S1 silicides are more easily formed at high temperature, while S2 silicides are more easily formed at low temperature.

### 3.7. Effect of Silicides on Creep Behavior

Silicon element has a significant effect on the creep behavior of TMCs. The solubility of Si element in α phase and β phase is different. During the creep process, due to temperature change and element diffusion, silicon element will further segregate to form silicides. Silicides limit grain boundary slip in the primary creep stage, while limit dislocation slips in the steady-state creep stage and accelerated creep stage [49]. The main creep mechanism of composites in this study is dislocation slip, so the strengthening mechanism of silicide creep behavior is its hindrance to dislocation movement. On the one hand, the pinning effect of fine silicides on dislocation is stronger than that of coarse silicides, and increased temperature and stress will coarsen silicide, resulting in the weakening of its strengthening effect on composites [17]. On the other hand, with the increase in temperature, silicon elements are more likely to form S1 silicides, and the short-rod-shaped S1 silicides have a better dislocation pinning effect than the spherical S2 silicides [50]. In addition, the size of silicides is also related to reinforcements. As shown in Figure 12f, due to the stacking fault structure of TiB whisker, the size of silicides formed near it is significantly larger than that around TiC and Y_2_O_3_ [51].

However, the strengthening effect of silicide on composites is limited. If the added Si is too little, the silicide will not be precipitated during the creep process, if the added Si is too much, the size of the silicide precipitated will increase and the non-uniformity will increase, thus weakening the strengthening effect. Even if an appropriate amount of Si element is added, as shown in Figure 8, Figure 9, Figure 10, Figure 11 and Figure 12, due to the difference in the solubility of Si in the α and β, the distribution of silicides in the material is uneven, so only a small part of the dislocations can be pinned, and the strengthening of the creep properties of the composite is very limited.

## 4. Conclusions

In this study, a (TiB + TiC + Y_2_O_3_)/α-Ti composite was prepared by induction skull melting. and then high temperature tensile creep experiments were conducted under different conditions. Detailed analysis of the effects of reinforcements and silicide on creep behavior and the main conclusions are summarized as follows:

1. The as-cast microstructure of the composite is a basket-weave structure, the main phase composition is lamellar α phase and a relatively low content of β phase, TiB is a whisker with large aspect ratio, TiC is equiaxed and mostly distributed near TiB, Y_2_O_3_ is micron-meter granular.
2. When the temperature increases from 650 °C to 700 °C, the steady-state creep rate of the composite increases by 1 to 2 orders of magnitude, and the creep life decreases significantly. After creep, TiB fractures and there is debonding between TiC and the matrix, while Y_2_O_3_ remains intact and has good bonding with the matrix.
3. The creep stress exponent of the composite at 650 °C and 700 °C is 2.92 and 2.96, respectively, indicating the main creep mechanism of this composite is dislocation slip. Temperature and stress have no significant effect on the creep mechanism of the composite.
4. The α/β interface has a hindering effect on the dislocation movement. With the increase in temperature or stress, the dissolution degree of the β phase increases, the α/β colonies are destroyed, and the limiting effect on the dislocation movement is weakened. Therefore, the increase of the dissolution degree of the β phase is one of the main reasons for the decrease of the creep life of the composite.
5. The reinforcements can improve composite structure, withstand loads, and hinder the dislocation movement during the creep process. Silicides precipitated near TiB and the α/β interface during creep can also restrict dislocation movement, thus reducing the creep rate and extending the creep life.

## Data Availability

The original contributions presented in this study are included in the article. Further inquiries can be directed to the corresponding author.
